# Filiform papillae do not have taste buds

**DOI:** 10.1590/S1808-86942012000400002

**Published:** 2015-10-20

**Authors:** Mehmet Fatih Sönmez, Esra Balcioglu, Saim Ozdamar, Birkan Yakan

**Affiliations:** Assistant Professor (Lecturer); MSc (Assistant); Professor (Lecturer)

**Keywords:** deafness, rats, taste buds, tongue

Numerous irregularities and mucosal bumps, known as tongue papillae, cover the dorsal surface of the anterior tongue region, all the way to the sulcus terminalis. There are four types of papillae, divided into: filiform, fungiform, circumvallate and foliated. Taste buds, which appear as oval shapes which look pale in histology sections, are present in the fungiform, foliated and circumvallate papillae, but not in the filiform^1^, ^2^, ^3^, ^4^.

We were very excited to read the recent paper from Abreu et al. called “Histological and ultra-structural aspects of the tongue in undernourished rats^5^”, showing that protein-energy malnutrition does not change the tongue mucosa of adult rats.

In the study from Abreu et al.^5^, vol. 72 $n^{\underline{o}}$ 4, p. 523-7, the legend in Figure 3 reads: “Fungiform and filiform papillae, over which are the taste buds”. However, filiform papillae do not have taste buds^6^. I would like to add that it may be useful to use arrows or asterisks to identify the structures in the pictures.
